# Stem signatures associating SOX2 antibody helps to define diagnosis and prognosis prediction with esophageal cancer

**DOI:** 10.1080/07853890.2022.2056239

**Published:** 2022-04-06

**Authors:** Zi-Yang Peng, Qing-Shi Wang, Kai Li, Si-Si Chen, Xiang Li, Guo-Dong Xiao, Shou-Ching Tang, Hong Ren, Zhe Wang, Xin Sun

**Affiliations:** a Department of Thoracic Surgery, the Second Department of Thoracic Surgery, Department of Thoracic Surgery and Oncology, Cancer Center, the First Affiliated Hospital of Xi’an Jiaotong University, Xi’an City, China; b Department of Pathology, Anatomy & Cell Biology, Sidney Kimmel Cancer Center, Thomas Jefferson University, Philadelphia, PA, USA; c Oncology Department, the First Affiliated Hospital of Zhengzhou University, Zheng Zhou City, China; d University of Mississippi Medical Center, Cancer Center and Research Institute, Jackson, MS, USA

**Keywords:** Stem cells signatures, SOX2, early diagnosis, oesophageal cancer, immune infiltration evaluation

## Abstract

**Background:**

esophageal cancer is one of the deadliest diseases worldwide. Due to the ineffectual screening methods referring to early diagnosis, most people have lost their chance of radical resection when diagnosed with esophageal cancer. This aim of this study was designed to evaluate the latent values of the stem signatures-associated autoantibodies (AABS) in predicting the early diagnosis, and particularly seeking the precise predictive outcomes with sensitive SOX2. We also studied the potential immunotherapeutic targets and prospective long-term prognosis predicators of esophageal cancer.

**Methods:**

The serum concentrations of selective antibodies were quantitated by enzyme-linked immunosorbent assay (ELISA), and a total of 203 local cases were enrolled. The TCGA databases were used to analyse distinct expression patterns and prognostic values of related genes. The TIMER database was used to explore the signatures of immune cell infiltration in related genes. The TISIDB database was used to analyse the association between related genes and immune regulators.

**Results:**

The stem signatures-associated with antibodies of TP53, PGP9.5, SOX2, and CAGE were highly expressed in esophageal cancer and were negatively correlated with the test group, the diagnostic sensitivity of P53, SOX2, PGP9.5 and CAGE reached to 54.3%, 56.5%, 80.4% and 47.8%, respectively, and the specificity reached 77.7%, 93.6%, 76.4% and 86.6%. Especially in stage I esophageal cancer, the diagnostic sensitivity of SOX2 reached 82.4% with a specificity of 85.4%, which demonstrated good value in early diagnosis.

**Conclusions:**

The stem signatures-associated antibodies could be used as an effective indicator in early esophageal cancer diagnosis and could help to precisely predicate survival and prognosis.

**Key Messages**
- The stem signatures-associated immune-antibodies could be used as effective indicators in early diagnosis of esophageal cancer and help to precisely predicate the survival and prognosis.
- The potential immunotherapeutic targets referring to esophageal cancer are screened and analysed, and the high sensitivity of SOX2 in detecting early esophageal cancer will yield early and effective treatments.

## 1. Introduction

esophageal cancer ranks seventh in terms of incidence and sixth in mortality overall, the latter index suggests that 1 in every 18 cancer deaths in the past 2020 was caused by esophageal cancer [1], which is one of the most aggressive digestive tracts of chest cancers. The overall five-year survival rate ranges from 15% to 25% worldwide and it is the sixth leading cause of cancer-related deaths of men [2]. esophageal cancer includes esophageal adenocarcinoma (EAC) and esophageal squamous cell carcinoma (ESCC), and the so-called Asian belt, which encompasses Turkey, northeastern Iran, Kazakhstan and northern and central China, has a very high incidence of esophageal SCC, with more than 100 cases per 100,000 population annually [3]. Most people diagnosed with esophageal cancer have lost their chance of surgery [1,2]. The previous diagnosis was mainly made by pathological biopsy under electronic endoscopy. Although some patients diagnosed with esophageal cancer are clinically cured by surgical treatment, the tumour is also prone to recurrence and metastasis within a certain period after relevant chemotherapy and targeted drugs. Intertumoral cancer cell heterogeneity contributes to therapy resistance in these cancers, so early diagnosis is indispensable in the treatment strategy of esophageal cancer [4]. Among them, the early and minimal invasive serological examination is a hot spot of current research, and related serum oncology biomarkers are considered as one of the early means to predict the occurrence of tumours [5–7].

Cancer stem-like cells, also known as cancer stem cells, cancer progenitor cells, were considered as the key roots for cancer occurrence and development, and their steady status made therapy insensible [3,5,8–10]. SOX2 is one of the most studied transcription factors and is critical for the digestive tract specification and regionalization, particularly in healthy organisms; it plays a role in stem cell regulation during embryogenesis, as well as during adult tissue regeneration, and also influences proliferation and apoptosis, as well as the migration and adhesion of cells [8,11]. The overexpression of SOX2 is frequently observed in the growth and propagation of tumours by clinical and experimental methods [8,12], which is currently considered as one of the main carcinogenic factors in the evolution of esophageal cancer.

Immune microenvironment and immune checkpoints blockade [13] have brought unprecedented hope for esophageal cancer patients who were considered as having lost the opportunity for radical surgery. Our study verify that cancer stem cells play an integral role in the immune microenvironment and are overexpressed in esophageal cancer, which suggests they may be used as potential immune checkpoints for relevant immunomodulatory targets [14].

Previous studies have demonstrated that autoantibodies are expected to be a new marker for the early diagnosis of esophageal cancer [15]. Recent serological studies have confirmed that cancer stem cells may be detectable several years before the development of symptomatic cancer and may serve as new screening markers for patients with cancer at the early stages [6]. We can use the results of early serological tests to predict whether patients have tumour-related risk and as a novel serological marker to screen for early esophageal cancer. For this purpose, we conducted a series of data analyses to study the value of stem cells in the diagnosis of early esophageal cancer, and to evaluate the prognosis of patients. As one of the largest thoracic oncology surgery centres in central and western China, it may help to identify potential patients with potentially early esophageal cancer and to improve a better prognosis.

## 2. Materials and methods

### 2.1. Clinical and pathological information

Based on strict inclusion criteria, a total of 203 patients from the central and western regions of China were admitted to the First Affiliated Hospital of Xi’an Jiaotong University, a Chinese regional medical centre, between 2016 and 2020, and all the patients were informed and agreed to participate in the relevant study. Among them, 46 patients were diagnosed with esophageal cancer by electronic endoscopic biopsy of digestive tract, and most of them were treated with surgical resection to further determine the tumour type and stage. Another 157 patients who confirmed benign lesions were all treated in the First Affiliated Hospital of Xi’an Jiaotong University for pneumonia, chest pain, intercostal neuritis and other related diseases during the same period as reference to set as a negative control group. The clinical data detail for all patients is shown in Table 1. Exclusion conditions: 1. The patient had never received prior chemotherapy, targeted therapy, immune-related drugs or surgical treatment; 2. Patients with known metastases to other organs and tumour stage T4; 3. There were primary tumours of other organs.

**Table 1.** Clinicopathological features of the 157 enrolled individuals.

Items	Esophageal carcinoma	Benign disease and healthy people
Gender		
Male	40	74
Female	6	83
Age		
≤60-year	13	111
>60-year	33	46
Smoking		
No	17	66
Yes	29	91
Drinking		
No	24	–
Yes	22	–
Histological subtype		
AD	10	–
SCC	36	–
TNM stage		
TI	30	–
TII	3	–
TIII	13	–

All patients were informed of the Helsinki Statement and the risks associated with the use of surgical excision of pathological specimens and signed an informed consent form. All study protocols and patients' informed consent were conducted under the authorization and supervision of the Ethics Committee of the First Affiliated Hospital of Xi’an Jiaotong University.

### 2.2. Enzyme-linked immunosorbent assay

The serum concentrations of immunology-related or generated autoantibodies were quantitated by enzyme-linked immunosorbent assay (ELISA). All subjects were drawn 10 ml of fasting venous blood. Centrifugation was applied immediately after the collection, and the upper serum was aspirated and stored. The autoantibodies were detected in strict accordance with the instructions of the kit (Kai Paul Company, Hangzhou). The absorbance of at least three independent groups at 450 nm was read and recorded [16].

### 2.3. Genes expression/mutations detections and clinical data analysis

Data of the mutation points and the mutation information were classified and filed in the Centre for Translation Medicine and the Centre Precision Medicine. Written information consents were obtained from all included patients. Detections were applied in either tumour cells, free plasma DNA or in archived paraffin-embedded tumour tissue, basins on different particularities. Samples were prepared and tested by using the next generation sequencing (NGS) of the Onco-Drug-Seq™ system (TOPGEN, Hangzhou, China). Both the single base variation of the gene and the small fragment insertion missing were tested referring to KRAS, TP53, EGFR. Specifically, detections included but were not limited to Exon-19, Exon-20, T790M, L858R, E709-T710delinsD, R108K, T263P, L747P, A750P, G12C and amplification.

ONCOMINE (https://www.oncomine.org/resource/login.html) [17] was used to analyse the gene expressions in NSCLC. The thresholds were restricted as follows: *p*-value = .05; fold-change = 1.5; gene rank = Top 10%. The expression levels of each gene in tumour specimens and normal tissues were compared. GEPIA (https://gepia.cancer-pku.cn) is an interactive web server for analysing the RNA sequencing expression data from The Cancer Genome Atlas (TCGA) and Genotype-tissue Expression (GTEx) dataset projects [18], which can reveal a concordance of the four members of antibodies with the surface markers of cancer stem cells. The databases detail is shown in Table 2. The database used for the correlation analysis was derived from Gene Expression Omnibus (GEO), GTEx, European Genome-phenome Archive (EGA) and TCGA [19].

**Table 2.** The concordance of the four members of stem signatures-associated antibodies with the surface markers of cancer stem cells.

ItemA	ItemB	Log2 odds ratio	*p*-value	q-value	Tendency
UCHL1	PROM1	>3	.056	0.293	Co-occurrence
UCHL1	ALDH1A1	>3	.141	0.370	Co-occurrence
TP53	CD44	1.424	.148	0.370	Co-occurrence
DDX53	PROM1	2.802	.176	0.370	Co-occurrence
DDX53	CD44	0.830	.470	0.626	Co-occurrence
SOX2	ALDH1A1	0.204	.608	0.709	Co-occurrence
SOX2	PROM1	>3	.039	0.270	Co-occurrence
SOX2	CD44	1.057	.297	0.480	Co-occurrence

### 2.4. Immune Response prediction statistical analysis

In this study, we explored different levels of immune cell infiltration in stem signatures-associated antibodies, which include Purity, B Cell, CD8+ T cell, CD4+ T cell, Macrophage, Neutrophil, Dendritic Cell expressions in esophageal cancer through the TIMER database [20]. Through the TISIDB database [21], we analysed the association between stem signatures-associated antibodies and Immuno-regulators, which included Immuno-inhibitors, Immuno-stimulator and MHC molecules.

### 2.5. Statistical analysis

Survival analysis was drafted using The Kaplan–Meier plotter [22]. Data are presented as the mean ± standard deviation (SD). The final conclusions of various antibody results were plotted into ROC curves. Mann–Whitney U test was used for mean comparison between two independent groups, and multiple independent samples were compared by Kruskal–Wills H test. The *p*-value less than .05 was considered statistically significant. All analyses were performed using the SPSS 24.0 statistical software (SPSS, Chicago, IL, USA) [16].

## 3. Results

### 3.1. The unique and conspicuous SOX2 overexpression indicated poor survival and recurrence expectance

**Figure 1.**
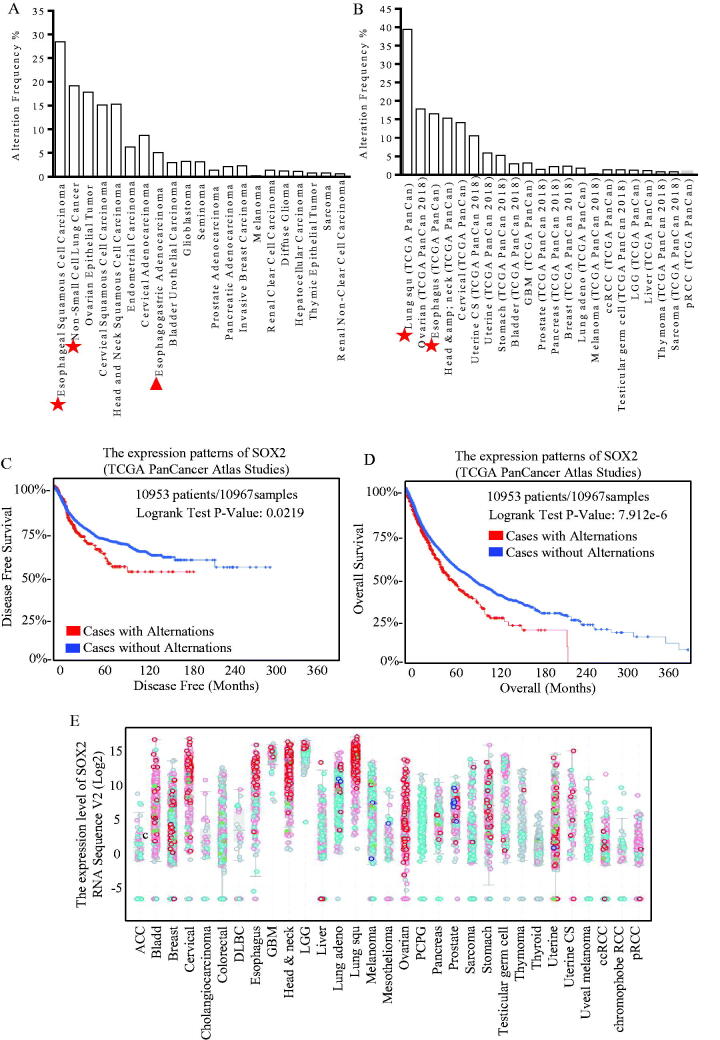
Different expression of SOX2 indicated diverse disease-free survival and overall survival expectations. Expression patterns of SOX2 in kinds of cancer were shown, and through TGCA database and heat map of genes, SOX2 is up-regulating expression in different tumour types, including esophageal, lung and ovarian cancer, especially in esophageal squamous carcinoma (A) and adenocarcinoma (B). The DFS and OS survival curves comparing patients with alteration (red) and without alteration (blue) referring to expression of SOX2 (C and D). Information on 10,953 patients and 10,967 samples were analysed through the TGCA Atlas database. We can know that cases with alternations have a lower disease free survival compared with cases without alternations (*p* = 0.0219, *p <.05), and cases with alternations have a much shorter overall survival compared with cases without alternations (*p* = 7.92e-6, *p<.05). Protein Atlas data were screened for SOX2 expression patterns (E). SOX2 upregulates the expression in different tumour types, including esophageal, head and neck, and lung squamous cancer.

The TCGA analysis (Pan-cancer) was performed and showed that SOX2 tended to be overexpressed more frequently in esophageal, lung and ovarian cancer, especially in esophageal squamous cell carcinoma, which was the common pathologic type in China(Figure 1(A,B)). Data from the TCGA Pan-Cancer Atlas Studies were used for survival analysis, and higher SOX2 expression correlated with much shorter disease-free survival (Figure 1(C)) and overall survival (Figure 1(d)). Protein Atlas data were screened for SOX2 expression patterns, and SOX2 overexpression was correlated with squamous esophageal carcinoma (Figure 1(E) and Figure S1(D)).

### 3.2. Sox2/notch signalling specific esophageal carcinoma phenotype

**Figure 2.**
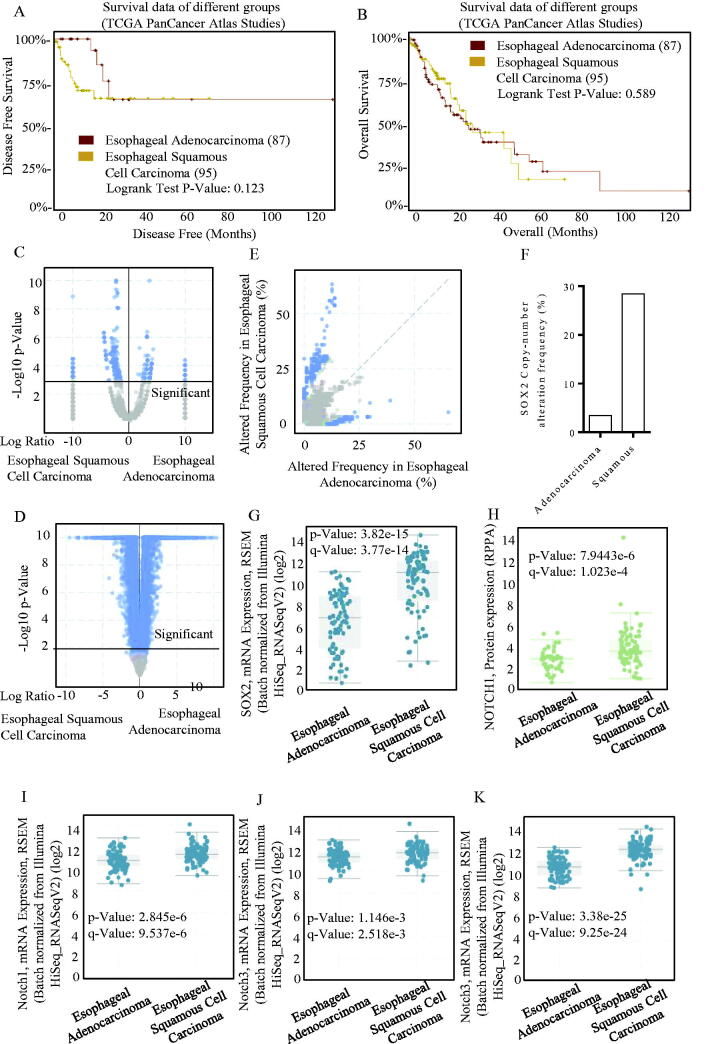
SOX2/Notch signalling phenotype in esophageal carcinoma. The OS and PFS survival curves comparing patients with esophageal adenocarcinoma (A) and esophageal squamous cell carcinoma (B) were plotted using Kaplan–Meier plotter analysis. (C–D) Different expression patterns of SOX2/Notch signalling phenotype in esophageal adenocarcinoma and esophageal squamous cell carcinoma were drafted, and the SOX2 expression patterns were much similar in either squamous carcinoma and adenocarcinoma. The overexpressed SOX2 was frequently occurred in either squamous carcinoma (E) and adenocarcinoma, especially in squamous esophageal carcinoma. (F) SOX2 expression level was significantly higher in squamous esophageal carcinoma than esophageal adenocarcinoma (G), and the SOX2-associated Notch signalling was co-activated (H–K). (I) The Notch1 signalling mRNA expression level was significantly higher in squamous esophageal carcinoma than esophageal adenocarcinoma. The Notch3 signalling mRNA expression level was significantly higher in squamous esophageal carcinoma than esophageal adenocarcinoma.

Stem cell potency-associated members were applied for expression identification using the CBIOPORTAL, and the profiled patterns of different studies are shown in Figure 2, which has emphasized a good concordance of the four members of PGP9.5, SOX2, TP53 and CAGE with the surface markers of cancer stem cells of either PROM1, CD44 or ALDH1A1. Overexpressed SOX2 did not result in survival differences in adenocarcinoma and squamous carcinoma (Figure 2(A,B)). SOX2 expression patterns were much similar in either squamous carcinoma and adenocarcinoma, and its overexpression was significant (Figure 2(C,D)). SOX2 expression signatures in squamous and adenocarcinoma were screened, and the overexpressed SOX2 was frequently occurred in squamous esophageal carcinoma (Figure 2(E,F)), indicating its cancer-type-specific existence. The SOX2 expression level was higher in squamous esophageal carcinoma (Figure 2(G)), and the SOX2-associated Notch signalling was co-activated (Figure 2(H–K)), which strongly suggested the squamous esophageal carcinoma-specific SOX2 signature. We found significant differences of these genes between cases with alternations and those without alternations through screening the database, and also, the decreased case numbers and median months were shown (Figure S1(A–C)). We also found that they interacted as upstream and downstream molecules (Figure S1(E)).

### 3.3. Sox2 predicated Asians-specific diagnosis and survival

**Figure 3.**
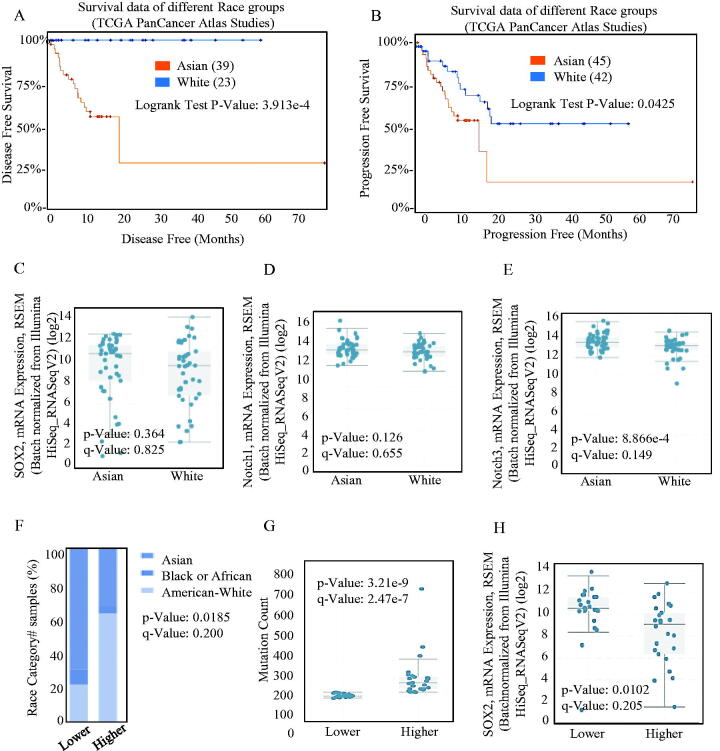
Different kind of expression of SOX2 in different race groups. The OS (A) and PFS (B) survival curves comparing patients with Asian (red) and White (blue) were plotted using Kaplan–Meier plotter scribing. Different mRNAs levels of SOX2/Notch signalling phenotypes in Asian and White were analysed, and the mRNA overexpression of SOX2 (C) and Notch1 (D) signalling was significant both in Asian and White. (E) The mRNA expression of Notch3 signalling was significantly different between Asian and White. (F) Asian patients always exhibited higher SOX2 alternation frequency than other patients. (G–H) Higher SOX2 alternation frequency was always significantly associated with higher mutation burden, and lower expression intensity.

The Kaplan–Meier analysis and log-rank test of The TCGA database both indicated that the increased SOX2 level was significantly associated with poorer overall survival (OS), indicating shorter disease-free survival (Figure 3(A)) and progression-free survival (Figure 3(B)) in Asian population. Asians diagnosed with ESCC tended to bear a higher SOX2 burden (Figure 3(C)), and the SOX2-associated Notch signalling was activated (Figure 3(D,E)). On the other hand, Asian patients often exhibited higher SOX2 alternation frequency (Figure 3(F)), which indicated better sensitivity and specificity. Higher SOX2 alternation frequency was always associated with higher mutation burden (Figure 3(G)), and lower expression intensity (Figure 3(H)).

### 3.4. Stem signatures-associated antibodies in immune response prediction diagnosis of esophageal cancer patients

**Figure 4.**
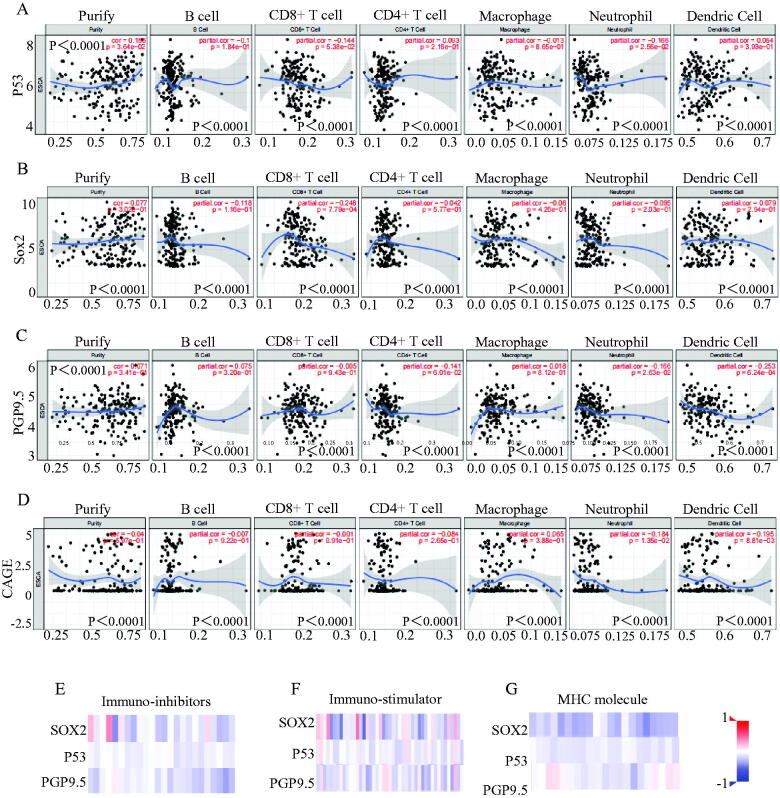
Stem signatures associated antibodies in immune response prediction diagnosis of esophageal cancer. The different levels of immune cell infiltration in stem signatures associated antibodies were analysed, including Purity, B Cell, CD8+ T cell, CD4+ T cell, macrophage, neutrophil, dendritic cell expressions in esophageal cancer through TIMER database. (A) P53 was associated with Purity (r = 0.156, *p* = 3.64e-02), B Cell (r= −0.1, *p* = 1.84e-01), CD8 + Tcell (r= −0.144, *p* = 5.38e-02), CD4 + Tcell(r = 0.093, *p* = 2.16e-01), Macrophage (r= −0.013, *p* = 8.65e-01), Neutrophil (r= −0.166, *p* = 2.56e-02), Dendritic Cell (r = 0.064, *p* = 3.93e-01). (B) SOX2 was significantly associated with Purity (r= −0.04, *p* = 5.97e-01), B Cell (r= −0.07, *p* = 9.22e-01), CD8 + Tcell (r= −0.001, *p* = 9.91e-01), CD4 + T cell (r= −0.084, *p*= *p* = 2.65e-01), Macrophage (r = 0.065, *p* = 3.88e-01), Neutrophil (r= −0.184, *p* = 1.35e-02), Dendritic Cell (r= −0.195, *p* = 8.81e-03). (C) PGP9.5 was significantly associated with Purity (r = 0.077, *p* = 3.02e-01), B Cell (r= −0.118, *p* = 1.16e-01), CD8 + Tcell (r= −0.248, *p* = 7.79e-04),CD4 + Tcell (r= −0.042, *p* = 5.77e-01), Macrophage (r= −0.06, *p* = 4.26e-01), Neutrophil (r= −0.095, *p* = 2.03e-01), Dendritic Cell (r = 0.079, *p* = 2.94e-01). (D) CAGE was significantly associated with Purity (r = 0.071, *p* = 3.41e-01), B Cell (r = 0.075, *p* = 3.20e-01), CD8 + Tcell (r= −0.005, *p* = 9.43e-01), CD4 + T cell (r= −0.141, *p* = 6.01e-02), Macrophage(r = 0.018, *p* = 8.12e-01), Neutrophil (r= −0.166, *p* = 2.63e-02), Dendritic Cell (r= −0.253, *p* = 6.24e-04). The results indicated that SOX2, P53, and PGP9.5 were negatively correlated to Immuno-inhibitors (E), immuno-stimulators (F), and MHC molecules (G).

We explored different levels of immune cell infiltration in stem signatures-associated antibodies and found that the stem signatures-associated antibodies expressions are correlated with immune infiltration levels in EC. We detected the correlation of P53 (Figure 4(A)), SOX2 (Figure 4(B)), PGP9.5 (Figure 4(C)), CAGE (Figure 4(D)) with levels of Purity, B Cell, CD8 + T cell, CD4+ T cell, macrophage, neutrophil, dendritic cell.

To further explore the effects of stem signatures-associated antibodies on tumour immune response, the correlations between the expression of stem signatures-associated antibodies and immuno-regulators were calculated. Related immune-inhibitors for ADORA2A, BTLA, CD160, CD244, CD274, CD96, CSF1R, CTLA4, HAVCR2, IDO1, IL10, IL10RB, KDR, KIR2DL1, KIR2DL3, LAG3, LGALS9, PDCO1, PDCD1LG2, PVRL2, TGFB1, TGFBR1, TIGIT, VTCN1 were selected for analysing in association with different expression in SOX2, P53 and PGP9.5. Related immune-stimulators for CD54, CD27, CD276, CD28, CD40, CD40, CD48, CD70, CD80, CD86, CXCL12, CXCR4, ENTPD1, HHLA2, ICOS, ICOSLG, IL2RA, IL6, IL6R, KLRC1, KLRK1, LTA, MICB, NT5E, PVR, RAET1E, TMEM173, TMIGD2, TNFRSF13B, TNFRSF13C, TNFRSF14, TNFRSF17, TNFRSF18, TNFRSF25, TNFRSF4, TNFRSF8, TNFRSF9, TNFSF13, TNFSF13B, TNFSF14, TNFSF15, TNFSF18, TNFSF4, TNFSF9 and ULBP1 were selected for analysing in different expression in SOX2, P53, and PGP9.5. Related MHC molecules for B2M, HLA-A, HLA-B, HLA-C, HLA-DMA, HLA-DMB, HLA-DOA, HLA-DOB, HLA-DPA1, HLA-DPB1, HLA-DQA1, HLA-DQA2, HLA-DQB1, HLA-DRA, HLA-DRB1, HLA-E, HLA-F, HLA-G, TAP1, TAP2 and TAPBP were selected for analysing in different expression in SOX2, P53 and PGP9.5. All relevant genes were significantly overexpressed in the immune microenvironment of esophageal cancer and interacted with multiple immune cells. The correlations between the expression of P53, SOX2, PGP9.5 and Immuno-inhibitors (Figure 4(E)), Immuno-stimulator (Figure 4(F)), and MHC molecules (Figure 4(G)) were calculated based on The TISIDB database. Further exploring the effects of stem signatures-associated antibodies on tumour immune response, P53, SOX2, and PGP9.5 are not only highly expressed in esophageal cancer patients, but also act as immune activators which regulate the immune system microenvironment and selectively action potential checkpoint blockade. Detail case numbers alternations were exhibited in supplemental data (Figure S1).

### 3.5. Diagnosis of esophageal cancer patients with stem signatures-associated antibodies

We performed a series of stratified grouping first divided into esophageal cancer patients, benign lesions and healthy people, always, our research has indicated that the signatures-associated antibodies related to the high-level expression may prompt early diagnosis of lung cancer, we applied to the determination of esophageal cancer patients, found that as shown, P53, PGP9.5, SOX2, CAGE, according to the results of quantitative of the signatures-associated antibodies in esophageal cancer-related levels also higher than that of benign disease and healthy people group.

**Figure 5.**
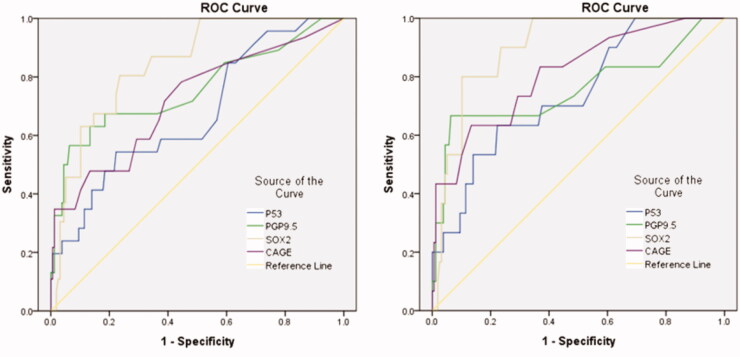
Diagnosis of esophageal cancer patients with Stem signatures associated antibodies. (A) ROC curves of four AAbs distinguishing esophageal cancer group from control groups. (B) ROC curves of four AAbs distinguishing esophageal squamous cell carcinoma cancer group from control groups (benign lesions and healthy people) (B).

The ROC curves (Figure 5(A)) showed that the AUC values of all antibodies were greater than 0.65 relatively, and specifically the AUC values of PGP9.5, SOX2 and Cage were greater than 0.7, indicating their high diagnostic value. According to the calculation formula, as was described in Table 3, the optimal cut-offs values were 3 U/ml for P53, 3.9 U/ml for PGP9.5,3.2 U/ml for SOX2, and 1.15 U/ml for CAGE. The diagnosis sensitivity of P53, SOX2, PGP9.5 and CAGE reached to 54.3%, 56.5%, 80.4%, and 47.8%, respectively, and the specificity reached to 77.7%, 93.6%, 76.4%, and 86.6%. We further analysed esophageal squamous cell carcinoma, benign lesions and healthy people and found that the AUC of each stem signatures-associated antibody was greater than 0.7, especially the SOX2 could reach 0.91 when his best cut-offs values were 4.55 U/ml for 91.7% and 85.4% of the diagnostic sensitivity and specificity for SOX2, which indicated a higher diagnostic efficiency.

**Table 3.** Diagnostic value of stem cell-associated antibodies in esophageal cancer and the control group.

		Area			Asymptotic 95% confidence interval		
Test result variable(s)		Area	Std. Error	Cut-offs	Lower bound		Upper bound
Dimension	P53	0.673	0.046	3	0.584		0.763
	PGP9.5	0.759	0.046	3.9	0.668		0.849
	SOX2	0.845	0.029	3.2	0.787		0.902
	CAGE	0.719	0.046	1.15	0.630		0.809

We also found that in early esophageal cancer patients, as was described in Table 4, the stem signatures-associated antibody indicators of SOX2 showed the specificity and sensitivity of 80% and 89.9% respectively in the ROC curve, when referring to stage I esophageal cancer patients (Figure 5(B)). Results demonstrate that as one of the esophageal serologic test indicators, SOX2 can not only better screen in high-risk groups, but also find the potential for patients with esophageal cancer. We performed a series of stratified grouping first divided into esophageal cancer patients, benign lesions and healthy people, always, our research has shown that the signatures-associated antibodies related to the high-level expression may prompt early diagnosis of lung cancer. We applied to the determination of esophageal cancer patients andfound that as shown, P53, PGP9.5, SOX2, CAGE, according to the results of quantitative of the signatures-associated antibodies in esophageal cancer-related levels also higher than that of benign disease and healthy people group, which shows that as one of the esophageal serologic test indicators, SOX2 can not only better screening in high-risk groups, but also finding the potential for patients with esophageal cancer (Table 5).

**Table 4.** Diagnostic value of stem cell-associated antibodies in esophageal cancer stage I and the control group.

Test result variable(s)		Area	Std. Error	Asymptotic 95% confidence interval	
				Lower Bound	Upper Bound
Dimension	P53	0.740	0.048	0.645	0.835
	PGP9.5	0.756	0.060	0.638	0.874
	SOX2	0.899	0.024	0.851	0.947
	CAGE	0.810	0.044	0.724	0.897

**Table 5.** The data of serum levels of TP53, PGP9.5, SOX2 and CAGE in esophageal cancer and the control group.

	TP53	PGP9.5	SOX2	CAGE
Average of EC	17.35938	8.48125	8.325	8.221875
Average of others	3.105882	1.263971	2.902941	0.775
Maximum value of EC	64.2	38.5	15.6	24.8
Maximum value of others	48.1	17.2	26.4	21.4
Minimum value of EC	0	0	0	0
Minimum value of others	0	0	0	0
Variance of EC	506.5043	116.6821	21.28688	86.44233
Variance of others	35.70511	5.705981	18.73911	5.547022

## 4. Discussion

Early diagnosis of esophageal cancer benefits the patients’ survival, and early treatments reduce the long-term complications and recurrence [23]. In the ongoing studies of early haematology detection indicators of esophageal cancer, research verifies that early tumours induce many tumours-related immune antibodies, which help us to detect esophageal cancer earlier by testing these stable and unique indicators of haematology.

In our previous studies, we found that stem signatures-associated antibodies can be used as a relevant indicator for screening of early stage lung cancer [24,25]. Through the analysis of the TCGA database in different styles, we discovered the highly expressed signatures in esophageal cancer patients, and also found that different human RACES have a different tendency in disease, so we further investigated the relevant cancer stem cell antibodies. The increased expression of SOX2 in esophageal cancer patients predicted a different outcome. Additionally, we found stem signatures-associated antibodies genes implied a greater immune system response, which provided a basis for us to investigate them as potential immune check suppression treatment sites. By further analysing data in patients with esophageal cancer, we found that cells harbouring high expression SOX2 expression do exist in the patients with esophageal cancer, and SOX2 itself could serve as a kind of serological indice used to detect whether patients lived and generated with esophageal cancer. Results are more credible in esophageal squamous carcinoma tissues, and grouping analysis showed SOX2 can be used as a kind of stem signatures-associated antibodies to detect early diagnosis of esophageal cancer occurrence, it may be through regulating NOTCH1, NOTCH2, ASCL4, FOXP1 pathways, influencing the tumour generation and progress [26–28]. Our research also confirmed the high sensitivity of SOX2 in detecting early esophageal cancer, and the sensitivity was 82.4%, with the specificity of 85.4%.

Patients with early esophageal cancer could be detected with sensitive SOX2 expression signature, and then treated with surgery as soon as possible, thus affecting the overall health level of patients. However, related issues are still worth exploring, for the number of cases in our centre is still less. Increasing the number of involving patients for statistical analysis, the refined prediction model could be achieved and used for erologic screening for the high-risk groups, identifying the early diagnosis.

## Supplementary Material

Supplemental Material

## Data Availability

The data that support the findings of this study are available from the corresponding author upon reasonable request.
