# Supplementary material for: Stem signatures associating SOX2 antibody helps to define diagnosis and prognosis prediction with esophageal cancer
Source: Ann Med. 2022 Apr 6;54(1):921–32. doi: 10.1080/07853890.2022.2056239 (PMC9004505; doi:10.1080/07853890.2022.2056239)

A

SOX2

Categories	Number of Cases	Decreased Cases	Median months
Cases with Alternations	338	88	NA
Without Alternations	5045	1011	NA

TP63

Categories	Number of Cases	Decreased Cases	Median months
Cases with Alternations	672	280	55.20
Without Alternations	10130	3234	81.63

B

NOTCH1

Categories	Number of Cases	Decreased Cases	Median months
Cases with Alternations	23	4	NA
Without Alternations	64	18	NA

NOTCH2

Categories	Number of Cases	Decreased Cases	Median months
Cases with Alternations	87	44	25.78
Without Alternations	95	32	28.11

C

ASCL4

Categories	Number of Cases	Decreased Cases	Median months
Cases with Alternations	39	18	17.59
Without Alternations	23	0	NA

FOXP1

Categories	Number of Cases	Progressed Cases	Median months
Cases with Alternations	45	21	15.68
Without Alternations	42	14	NA

D

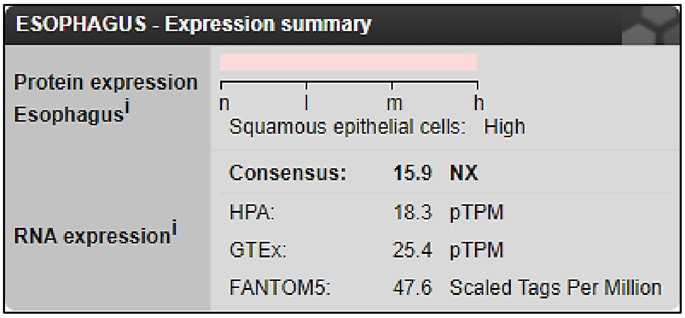

E

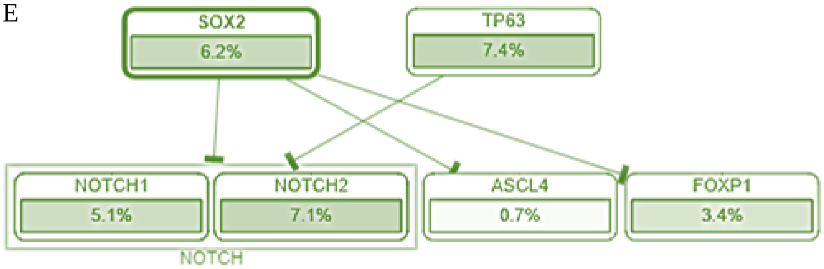

Supplement: Supplemental Material [file IANN_A_2056239_SM9050.zip › Supplemental files/Figure S1.pdf]
