# Supplementary material for: Stem signatures associating SOX2 antibody helps to define diagnosis and prognosis prediction with esophageal cancer
Source: Ann Med. 2022 Apr 6;54(1):921–32. doi: 10.1080/07853890.2022.2056239 (PMC9004505; doi:10.1080/07853890.2022.2056239)
Supplement: Supplemental Material [file IANN_A_2056239_SM9050.zip › Supplemental files/Supplementary Material.docx]

Stem signatures associating SOX2 antibody helps to define diagnosis and prognosis prediction with Esophageal cancer

Zi-Yang Peng^1^, Qing-Shi Wang^1^, Kai Li^1^ , Si-Si Chen^1^, Xiang Li^1,2^, Guo-Dong Xiao^3^, Shou-Ching Tang^4^, Hong Ren^1^, Zhe Wang^1,*^ Xin Sun^1,*^

^1^ Department of Thoracic Surgery, the Second Department of Thoracic Surgery, Department of Thoracic Surgery and Oncology, Cancer Center, the First Affiliated Hospital of Xi’an Jiaotong University, Xi’an City, Shaanxi Province, 710061, China;

^2^ Department of Pathology, Anatomy & Cell Biology, Sidney Kimmel Cancer Center, Thomas Jefferson University, Philadelphia, PA 19107, USA;

^3^ Oncology Department, the First Affiliated Hospital of Zhengzhou University, Zheng Zhou City, Henan Province, 450052, China;

^4^ University of Mississippi Medical Center, Cancer Center and Research Institute, 2500 North State Street, Jackson, Mississippi, 39216, USA.

* Correspondence should be addressed to:

1. Xin Sun, M.D., Research Associate, Attending Physician, Department of Thoracic Surgery, Department of Thoracic Surgery and Oncology, Cancer Center, the First Affiliated Hospital of Xi’an Jiaotong University, 277 Yanta West Road, Xi’an, Shaanxi Province, 710061, China. E-mail: [dr_sun_endeavour@163.com](mailto:dr_sun_endeavour@163.com), [dr_xinsun_87@xjtu.edu.cn](mailto:dr_xinsun_87@xjtu.edu.cn), Tel: (86) 18220572193; Fax: 86 (029)-85323473.

2. Zhe Wang, M.D., Associate Professor, Department of Thoracic Surgery, the Second Department of Thoracic Surgery, Department of Thoracic Surgery and Oncology, Cancer Center, the First Affiliated Hospital of Xi’an Jiaotong University, 277 Yanta West Road, Xi’an, Shaanxi Province, 710061, China. E-mail: wangzheradish@sohu.com, Tel: (86) 18991232615; Fax: 86 (029)-85323473.

**Supplemental Figure Legends**

Figure S1 The median months of involving cases with or without alteration referring to protein and RNA expression signatures in esophageal cancer

A. The difference of SOX2 and TP63 gene in Number of Cases, Decreased cases and Median months in Cases with Alternations and Without Alternations. B. The difference of NOTCH1 and NOTCH2 gene in Number of Cases, Decreased cases and Median months in Cases with Alternations and Without Alternations. C. The difference of ASCL4 and FOXP1 gene in Number of Cases, Decreased cases and Median months in Cases with Alternations and Without Alternations. D. The protein and RNA indices high expression in Esophageal cancer. E. The interaction in SOX2, TP63, NOTCH1, NOTCH2, ASCL4 and FOXP1.
